# Clinical and neuroimaging insights into childhood arterial ischemic stroke of different ages

**DOI:** 10.3389/fneur.2025.1568684

**Published:** 2025-04-04

**Authors:** Guanhao Liu, Qiang Ding, Bohao Wang, Qianhong Hu, Mengmeng Sun, Yafeng Liang, Qianlei Zhao

**Affiliations:** ^1^Department of Pediatric Neurology, The Second Affiliated Hospital and Yuying Children’s Hospital of Wenzhou Medical University, Zhejiang, China; ^2^The Second School of Medicine of Wenzhou Medical University, Wenzhou, China; ^3^Department of Neonatology, The Second Affiliated Hospital and Yuying Children’s Hospital of Wenzhou Medical University, Wenzhou, China; ^4^Department of Medical Imaging, The Second Affiliated Hospital and Yuying Children’s Hospital of Wenzhou Medical University, Wenzhou, China; ^5^Department of Pediatric Emergency, The Second Affiliated Hospital and Yuying Children’s Hospital of Wenzhou Medical University, Wenzhou, China

**Keywords:** stroke, children, arterial ischemic stroke, clinical manifestations, neuroimaging, risk factors

## Abstract

**Background:**

Childhood stroke is a neurological emergency and an important cause of acquired brain injury and mortality in children. This retrospective study aimed to investigate the clinical presentation and neuroimaging features of arterial ischemic stroke (AIS) in children.

**Methods:**

We retrospectively reviewed the records of pediatric AIS including neonates and children under 18 years of age from January 2008 to December 2023. Then we analyzed the gender and age distribution of AIS, as well as the clinical and neuroimaging characteristics and risk factors of AIS in different age groups. The study was approved by the Ethics Committee of the host institution.

**Results:**

Male patients predominated in both the neonatal AIS (NAIS) group and the pediatric AIS (PAIS) group. The majority of AIS children (73, 60.33%) were diagnosed after 24 h of symptom onset. Seizures (82.35%) and limb weakness (77.88%) were the most common initial neurologic symptoms of NAIS and PAIS, respectively. Anterior circulation AIS alone was much more common than posterior circulation AIS alone in childhood AIS (79.34% vs. 9.92%). However, the NAIS group had a higher rate of infarctions that solely involved the cortex (52.94% vs. 20.19%). Perinatal hypoxia or asphyxia (23.5%) and minor head injury (28.85%) were the most common possible risk factors in NAIS and PAIS, respectively.

**Conclusion:**

AIS in children is male-predominant, and there is significant diagnostic delay in both NAIS and PAIS. NAIS and PAIS differ in clinical and neuroimaging manifestations, and risk factors. Notably, we also noted that the actual prevalence of AIS in children, and the diagnosis of certain risk factors, may be underestimated. Education and training will be necessary in both layperson and healthcare settings. Furthermore, prospective studies are required to explore this hypothesis.

## Introduction

Stroke remains one of the greatest global health challenges (1). Childhood stroke is a neurological emergency and an important cause of acquired brain injury and mortality in children. Stroke in children is relatively rare compared to that in adults, and its incidence rate varies across countries with different economic levels. The global incidence of stroke in children and adolescents has shown an increasing trend, with a global estimated incidence of approximately 2–4.6 cases per 100,000 person-years, or even higher (2–4). Stroke in children is often under-recognized and delayed in diagnosis, leading to lifelong disability and even death. It is well known that deficits in neonates and children can only be diagnosed once certain milestones are reached, and these deficits often have a greater impact not just on the child but also on the family as children grow with their deficits. More than half of survivors are likely to experience persistent neurological deficits, including varying degrees of motor or intellectual impairment, which have profound effects on psychosocial functioning and quality of life (5–7).

Arterial ischemic stroke (AIS) is defined as focal brain damage and acute neurological deficit lasting more than 24 h due to cerebral vasospasm, stenosis or occlusion. Globally, ischemic stroke is the predominant type of stroke among all incident strokes, accounting for 62.4% (8), while half of all strokes in children are ischemic and half are hemorrhagic (9). AIS in children is not only very different from that in adults in terms of prevalence, but also in terms of risk factors, clinical course and prognosis (1). AIS can occur at all age stages in childhood. Neonatal arterial ischemic stroke (NAIS) refers to AIS in newborns within 28 days of birth. Pediatric arterial ischemic stroke (PAIS) is defined as AIS in non-neonatal children between 29 days and 18 years of age (10). AIS in children of different age stages also has different clinical presentation and risk factors. Newborns with AIS may have no specific clinical manifestations at birth. Seizures are often the primary clinical manifestation of NAIS and typically present as focal motor seizures (3, 11). NAIS often develop motor or cognitive dysfunction only a few months after birth, making early diagnosis difficult (12). The etiology and risk factors of childhood AIS varies considerably among different ethnic groups and regions. Previously reported, the most common causes of childhood AIS in western countries were sickle cell disease (SCD) and congenital or acquired heart disease (13). However, a retrospective analysis from a medical center in southwest of China suggests that minor head injury-related stroke is the main risk factor of PAIS (5).

The purpose of this study was to retrospectively analyze the clinical spectrum, neuroimaging features of childhood AIS aged 0–18 years in our medical center and to evaluate possible risk factors of these patients.

## Participants and methods

The study was approved by the Ethics Committee of the Second Affiliated Hospital of Wenzhou Medical University (approval number: 2024-K-176-01). This study was a retrospective analysis that did not involve human specimens or any personal information. It was approved by the Ethics Committee, which waived the need for informed consent.

### Patients

**Figure 1:**
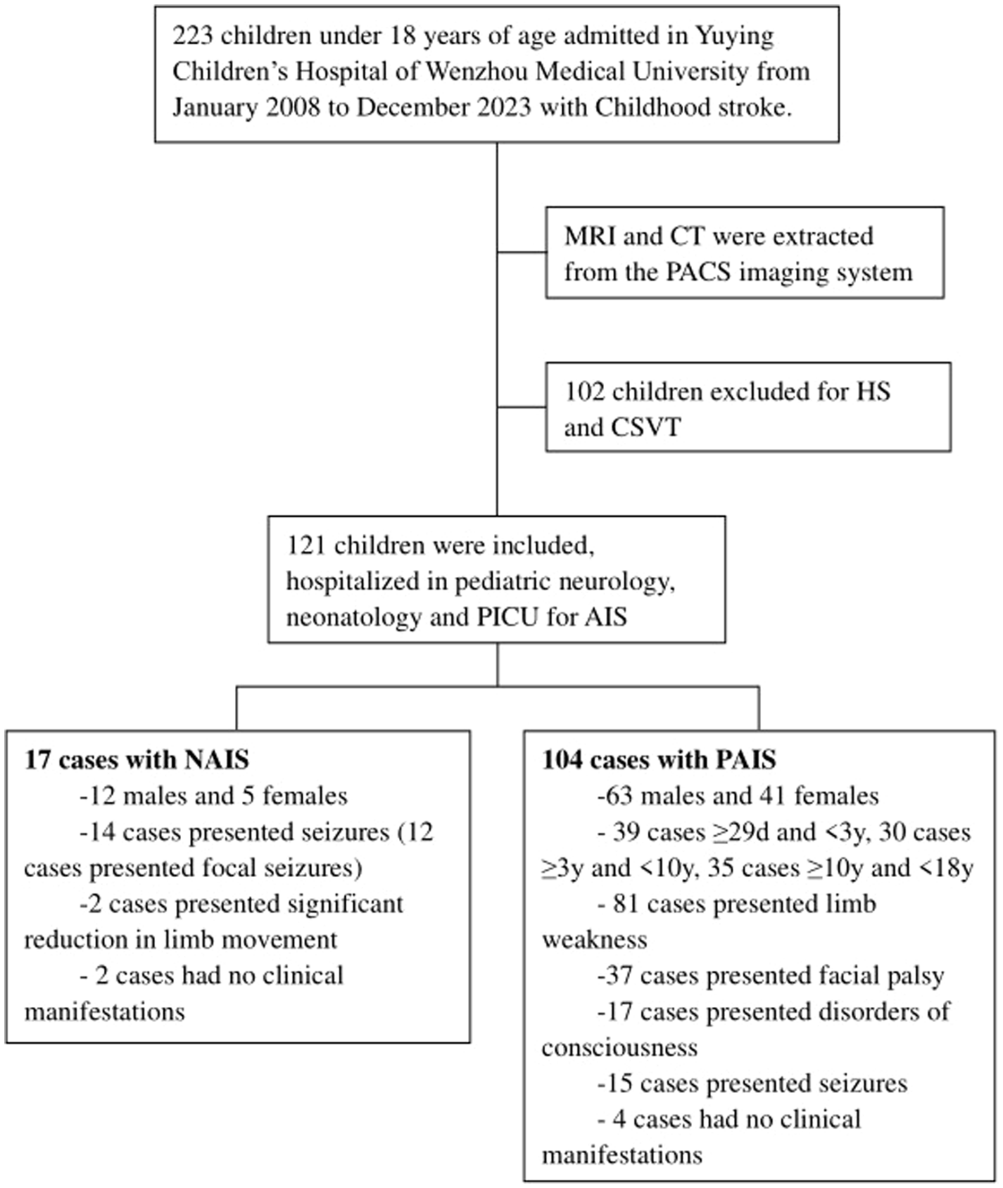
Study flowchart. MRI, magnetic resonance imaging; CT, computed tomography; PACS, picture archiving and communication systems; HS, hemorrhagic stroke; CSVT, cerebral sinus venous thrombosis; PICU, pediatric intensive care unit; AIS, arterial ischemic stroke; NAIS, neonatal arterial ischemic stroke; PAIS, pediatric arterial ischemic stroke.

In this study, we retrospectively reviewed the records of childhood AIS including neonates and children under 18 years of age in Yuying Children’s Hospital of Wenzhou Medical University, a comprehensive pediatric medical center in eastern China, from January 2008 to December 2023 (Figure 1). The inclusion criteria were as follows: (i) Neonates and children under 18 years of age. (ii) Presence of acute focal neurological deficit lasting more than 24 h, with confirmation of AIS by computed tomography (CT) or magnetic resonance imaging (MRI). (iii) Even in the absence of clinical manifestations, cases with newly diagnosed AIS confirmed by MRI and vascular imaging were also included in this study. The exclusion criteria were as follows: (i) Age over 18 years. (ii) Intracerebral hemorrhage or subarachnoid hemorrhage confirmed by CT or MRI were excluded.

### Data collection

We collected population characteristics of patients, including gender and age at onset. The children were grouped according to their age. AIS within 28 days as the NAIS group, AIS between 29 days and 18 years of age as the PAIS group. PAIS was categorized into three groups based on age. We also collected data on clinical presentation, time to diagnosis after the onset of clinical symptoms, medical history, and laboratory test results to determine risk factors. Brain images of all children with AIS, including MRI and CT, were retrospectively extracted from the PACS imaging system and analyzed for stroke size, infarct localization. All infarcts with a maximum diameter measuring less than 1.5 cm were classified as small (14). In children with anterior circulation AIS, retrospective Alberta Stroke Program Early CT Score (CT-ASPECTS) or diffusion-weighted imaging-ASPECTS (DWI-ASPECTS) scores were used for semiquantitative assessment of early ischemic lesions (1, 15–17). Briefly (1, 17), two standard axial cuts and 10 regions of the middle cerebral artery (MCA) supply were selected at CT or MRI with DWI. One standard axial cut at the level of the thalamus and basal ganglia, containing anterior cortical area part frontal lobe (M1), cortical area lateral to island ribbon (M2), posterior cortical area temporal lobe (M3), island (I), lenticular nucleus (L), caudate nucleus (C) and inner capsule (IC) 7 regions. Another standard axial cut just rostral to the ganglionic structures, above the thalamus and basal ganglia, containing anterior area located above M1 area (M4), central area located above M2 area (M5) and posterior area located above M3 area (M6) 3 regions. These 10 regions were given the same weighting of 1 point. 1 point was subtracted for the presence of early ischemic changes in each defined region, such as focal swelling or hypoattenuation of parenchymal tissue on CT or signal changes on DWI. The ASPECTS or DWI- ASPECTS value was calculated from these 10 regions. A score of 10 is normal, and a score of 0 indicates diffuse infarction in supplying territory of the MCA.

### Statistical analysis

SPSS 23 software was used for statistical analysis. Descriptive data were recorded as numbers and frequencies. Continuous data were described by means and standard deviation (means ± SD). Chi-square test, including Fisher exact or Pearson *χ*^2^, was conducted for categorical variables. *t* test and One-way analysis of variance (ANOVA) were used for continuous variables. *p* value < 0.05 was considered to be statistically significant.

## Results

### Population characteristics

A total of 121 AIS children were included in our retrospective analysis. Among them, 75 were males (61.98%) and 46 were females (38.02%). The age of the patients ranged from day 1 of life to 17.9 years. There were 17 patients (14.05%) in NAIS group, 12 males (70.59%) and 5 females (29.41%), with a mean age at onset of 1.4 ± 1.6 days, of which the exact age at onset could not be determined in 2 cases due to the absence of clinical symptoms. There were 104 patients (85.95%) in PAIS group, 63 males (60.58%) and 41 females (39.42%). Both the NAIS and PAIS groups were male predominance. There was more PAIS than NAIS, which we believed was partly due to our hospital serving as a regional referral center, resulting in more PAIS cases being referred from other hospitals, and partly due to the larger population base of children compared to newborns. There was no statistically significant difference between the two groups in terms of sex ratio (*χ*^2^ = 0.621, *p* = 0.430). In the PAIS group, the mean age was 6.6 ± 5.4 years (6.24 ± 5.54 years for males and 7.12 ± 5.26 years for females), with no statistical difference in age between genders (*t* = 0.813, *p* = 0.418). The gender distribution and mean age by gender of the PAIS group at different age stages were shown in Table 1 and no statistically significant differences were found in the age of onset by gender across age groups (The *t* values and *p* values for the three groups were *t* = 0.633, *p* = 0.53, *t* = 0.37, *p* = 0.76 and *t* = 0.266, *p* = 0.792 respectively).

**Table 1:** Gender distribution and mean age by gender in PAIS across age groups.

Age groups	*n* (%)		Ages (years)	
	Males	Females	Males	Females
≥29 d, <3 *y* (*n* = 39)	28 (71.79)	11 (28.21)	1.20 ± 0.75	1.37 ± 0.71
≥3 y, <10 y (*n* = 30)	14 (46.67)	16 (53.33)	5.76 ± 2.14	5.53 ± 1.98
≥10 y, <18 y (*n* = 35)	21 (60.00)	14 (40.00)	13.27 ± 2.00	13.46 ± 2.39
Total (*n* = 104)	63 (60.58)	41 (39.42)	6.24 ± 5.54	7.12 ± 5.26

### Clinical manifestations

More than half of AIS children (73, 60.33%) were diagnosed after 24 h of symptom onset (76.4% in NAIS vs. 57.69% in PAIS, *χ*^2^ = 2.153, *p* = 0.142), and only 11.54% of PAIS were diagnosed within 6 h of the onset of clinical symptoms (Table 2). Four patients in PAIS group were unable to determine the exact time of infarction, of which 3 patients were asymptomatic and one patient was detected as a failure to regain consciousness postoperatively. Sixty cases (57.69%) of PAIS diagnosed after 24 h of symptom onset were further analyzed, of which half of them (28 cases, 26.92%) presented to the hospital with symptoms that had persisted for more than 24 h. Two patients in NAIS group were unable to determine the exact time of infarction due to the absence of clinical symptoms.

**Table 2:** Diagnostic delay in NAIS and PAIS, *n* (%).

Diagnostic delay, h	NAIS (*n* = 17)	PNIA (*n* = 104)	Total (*n* = 121)
≤6 h	0 (0.00)	12 (11.54)	12 (9.92)
>6, ≤12 h	2 (1.92)	12 (11.54)	14 (11.57)
>12, ≤24	0 (0.00)	16 (15.38)	16 (13.22)
>24 h	13 (76.4)	60 (57.69)	73 (60.33)
Unknown	2 (1.92)	4 (3.84)	6 (4.96)

**Figure 2:**
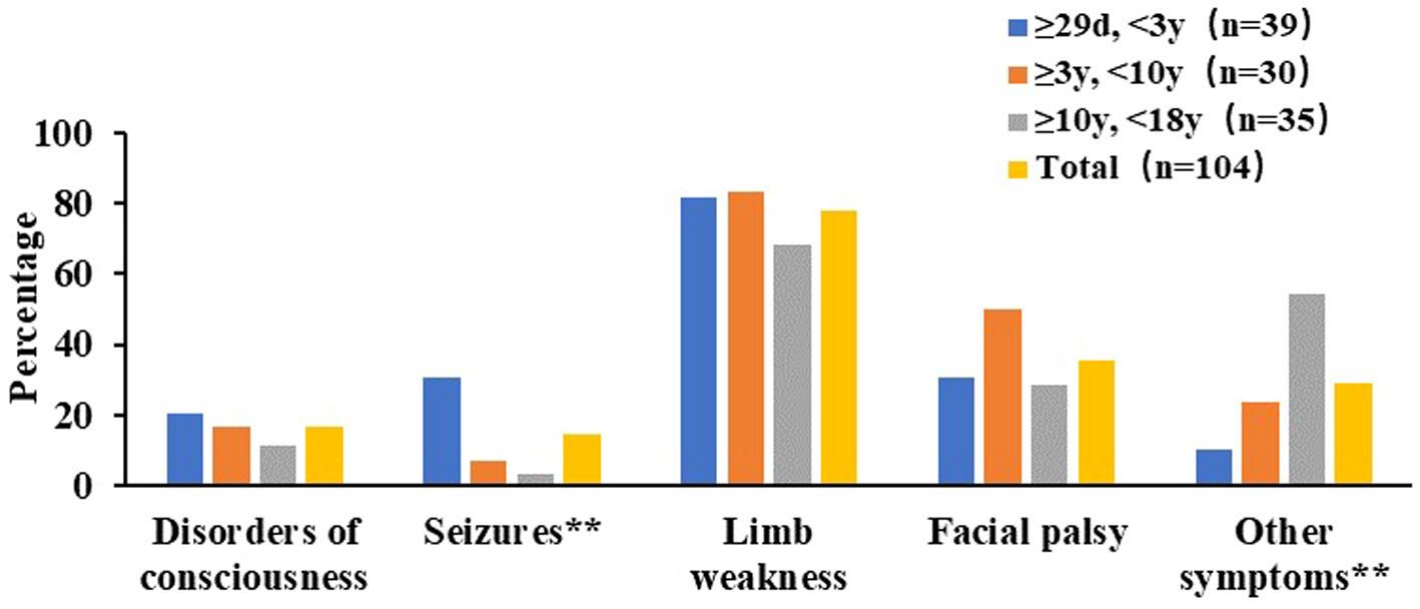
Nervous system symptoms at initial presentation of in PAIS. ** Chi-square test had significant difference among different age groups in PAIS, *P* < 0.01.

In NAIS group, seizures were the most common initial neurologic symptoms, with 14 cases (82.35%) of NAIS presenting with seizures. Of these, 12 cases (85.71%) presented focal seizures, which were the predominant form. Only 2 cases (1.92%) of NAIS presented significant reduction in limb movement. Of these 2 cases, 1 was accompanied by seizure and 1 showed only reduction in limb movement. Two cases (1.92%) of NAIS had no clinical manifestations. In PAIS group, limb weakness (81 cases, 77.88%), facial palsy (37 cases, 35.58%), disorders of consciousness (17 cases, 16.35%), and seizures (15 cases, 14.42%), were the main initial neurologic symptoms of PAIS (Figure 2). Ninety-two cases (88.46%) of PAIS presented with these 4 symptoms. Seizures were significantly less prevalent in PAIS than in NAIS (14.42% vs. 82.35%, *χ*^2^ = 33.366, *p* < 0.01) and occurred mainly under 3 years of age. Other neurologic-related symptoms, including headache (12 cases, 11.54%), vomiting (13 cases, 12.50%), aphasia or dysarthria (7 cases, 6.73%), unsteady gait (2 cases, 1.92%), blurred vision (1 cases, 0.96%), and ptosis (1 cases, 0.96%), and these symptoms seem to be more prominent in the group of children over the age of 10 years. In addition, 89 cases (85.58%) of PAIS had at least 2 or more symptoms.

### Neuroimaging findings

Eleven cases (64.7%) of NAIS infarctions were located on the left side. Left-sided infarctions were slightly higher than right-sided in PAIS (47.11% vs. 45.19%) (Table 3), with no statistical difference between the two groups (*χ^2^* = 1.083, *p* = 0.298). Large stroke size predominated in both groups (82.35% vs. 74.04%, *χ^2^* = 0.188, *p* = 0.665). Anterior circulation AIS only were much more common than posterior circulation AIS only in childhood AIS (96 cases, 79.34% vs. 12 cases, 9.92%). However, the NAIS group had a higher rate of infarctions that solely involved the cortex (52.94% vs. 20.19%), with a statistically significant difference (*χ^2^* = 6.740, *p* < 0.01). CT-ASPECTS or DWI- ASPECTS scores were used for semiquantitative assessment of early ischemic lesions in anterior circulation AIS retrospectively. Scores greater than 7 were 39 cases (40.63%) in anterior circulation AIS. The values indicated no significant difference between the NAIS and PAIS groups (5.64 ± 2.59 vs. 5.91 ± 2.93, *t* = 0.326, *p* = 0.745). There was also no statistical difference among the different age groups in the PAIS group (*F* = 0.204, *p* = 0.816).

**Table 3:** Neuroimaging findings of NAIS and PAIS, *n* (%) or (means ± SD).

Neuroimaging findings	NAIS	PAIS			
		≥29 d, <3 y (*n* = 39)	≥3 y, <10 y (*n* = 30)	≥10 y, <18 y (*n* = 35)	Total (*n* = 104)
Side					
Left	11 (64.71)	19 (48.72)	15 (50.00)	15 (42.86)	49 (47.11)
Right	6 (35.29)	16 (41.03)	13 (43.33)	18 (51.43)	47 (45.19)
Bilateral	0 (0.00)	4 (10.26)	2 (6.67)	2 (5.71)	8 (7.69)
Stroke size					
Small	3 (17.65)	12 (30.77)	3 (10.00)	12 (34.4)	27 (25.96)
Large	14 (82.35)	27 (69.23)	27 (90.00)	23 (65.7)	77 (74.04)
Circulation and infarct localization					
**Anterior circulation**	14 (82.35)	33 (84.62)	23 (76.67)	26 (74.29)	82 (78.85)
Basal ganglia	4 (23.53)	18 (46.15)	9 (30.00)	9 (25.71)	36 (34.62)
Cortex not basal ganglia**	9 (52.94)	6 (15.38)	7 (23.33)	8 (22.86)	21 (20.19)
Cortex and basal ganglia	1 (5.88)	9 (23.08)	7 (23.33)	9 (25.71)	25 (24.04)
ASPECTS (mean)	5.64 ± 2.59	5.69 ± 3.29	5.91 ± 2.57	6.19 ± 2.81	5.91 ± 2.93
ASPECTS (>7)	5 (35.71)	17 (51.52)	8 (34.78)	9 (31.03)	34 (41.46)
**Posterior circulation**	0 (0.00)	1 (2.56)	3 (10.00)	8 (22.86)	12 (11.54)
Thalamic	0 (0.00)	0 (0.00)	1 (3.33)	3 (8.57)	4 (3.85)
Cerebellar	0 (0.00)	1 (2.56)	1 (3.33)	3 (8.57)	5 (4.81)
Brainstem	0 (0.00)	0 (0.00)	0 (0.00)	2 (5.71)	2 (1.92)
Other	0 (0.00)	0 (0.00)	1 (3.33)	0 (0.00)	1 (0.96)
**Anterior and posterior circulation**	3 (17.65)	5 (12.82)	4 (13.33)	1 (2.86)	10 (9.62)

**Chi-square test had significant difference between NAIS and PAIS, *P* < 0.01.

### Risk factors analysis

There were numerous causes of AIS, and possible risk factors were present in 13 cases (74.47%) in the NAIS group and 82 cases (78.85%) in the PAIS group. Risk factors were significantly different between the two groups (Tables 4, 5). In NAIS group, perinatal hypoxia or asphyxia (4 cases, 23.53%) was the most common possible risk factor in the NAIS group, while minor head injury (30 cases, 28.85%) was the most common risk factor in the PAIS group, followed by heart disease (14 cases, 13.46%), cerebrovascular malformations (12 cases, 11.54%), and intracranial infections (9 cases, 8.65%). Of note, 80% of minor head injuries occurred in children under 3 years of age, decreasing with age. Among them, 10 cases (33.33%) were accidental falls and 7 cases (23.33%) were bed falls. Of the 12 cases of cerebrovascular malformations, 5 cases (41.67%) were moyamoya disease. Of the 14 cases of heart disease, 5 cases (35.71%) had structural malformations of the heart, 4 cases (28.57%) were infective endocarditis, 3 cases (21.43%) were cardiomyopathy, 1 case (7.14%) was arrhythmia, and 1 case (7.14%) was myocarditis with thrombosis. Of the 9 cases of intracranial infections, 4 cases (44.44%) were diagnosed with mycoplasma encephalitis, 3 cases (33.33%) with viral encephalitis, 1 case (11.11%) with tuberculous meningitis, and 1 case (11.11%) with brain abscess. In addition, 11 cases (10.58%) of PAIS had at least 2 risk factors.

**Table 4:** Risk factors of NAIS, *n* (%).

Risk factors	NAIS (*n* = 17)
Perinatal hypoxia or asphyxia	4 (23.53)
Infectious disease	1 (5.88)
Surgery	1 (5.88)
Abnormal fetal position	1 (5.88)
Premature birth	1 (5.88)
Heart disease	1 (5.88)
Fetal macrosomia	1 (5.88)
Birth injury	1 (5.88)
Twin pregnancy	1 (5.88)
Electrolyte disturbance	2 (11.76)
Undefined	4 (23.53)

**Table 5:** Risk factors of PAIS, *n* (%).

Risk factors	≥29 d, <3 y (*n* = 39)	≥3 y, <10 y (*n* = 30)	≥10 y, <18 y (*n* = 35)	Total (*n* = 104)
Minor head injury**	24 (61.54)	5 (16.67)	1 (2.86)	30 (28.85)
Heart disease	3 (7.69)	5 (16.67)	6 (17.14)	14 (13.46)
Cerebrovascular malformations	2 (5.13)	4 (13.33)	6 (17.14)	12 (11.54)
Intracranial infections	2 (5.13)	5 (16.67)	2 (5.71)	9 (8.65)
Systemic infectious diseases	0 (0.00)	4 (13.33)	2 (5.71)	6 (5.77)
Surgery	3 (7.69)	1 (3.33)	1 (2.86)	5 (4.81)
Metabolic disease	1 (2.56)	0 (0.00)	4 (11.43)	5 (4.81)
Acute hypoxia or asphyxia	1 (2.56)	0 (0.00)	0 (0.00)	1 (0.96)
Hypertension	1 (2.56)	0 (0.00)	0 (0.00)	1 (0.96)
Intracranial mass	0 (0.00)	4 (13.33)	0 (0.00)	4 (3.85)
Pulmonary artery stenosis	0 (0.00)	0 (0.00)	2 (5.71)	2 (1.92)
Hematologic disorder	1 (2.56)	0 (0.00)	0 (0.00)	1 (0.96)
Idiopathic vasculitis	0 (0.00)	0 (0.00)	1 (2.86)	1 (0.96)
Renal disease	1 (2.56)	0 (0.00)	1 (2.86)	2 (1.92)
Undefined	2 (5.13)	6 (20.00)	14 (40.00)	22 (21.15)

** Chi-square test had significant difference among different age groups in PAIS, *p* < 0.01.

## Discussion

AIS has a low prevalence in children, but it is an important etiologic factor contributing to severe neurologic consequences. The literature has reported a higher incidence of stroke in males than in females at all ages (18). Several surveys have also shown gender differences in childhood AIS. An international pediatric stroke study showed a male predominance in childhood AIS (61% for neonates, 59% for later childhood) (19). Xie et al., retrospectively analyzed PAIS in Southwest China, and the results suggested that 70.4% were male and 29.6% were female (5). deVeber et al.’s analysis of age-specific epidemiological characteristics of NAIS and PAIS also showed a male predominance (55%) of childhood AIS (20). Our results showed gender differences in childhood AIS, with males accounting for 70.59% in NAIS and 60.58% in PAIS. Both the NAIS and PAIS groups were male-predominant. Studies in adults have shown that the age of onset of stroke is generally older in females compared to males (18, 21, 22). We compared the age of onset of PAIS in different age stages and no statistically significant differences were found in the age of onset by gender across age groups.

Diagnostic delay is a common problem with AIS in children. The median time from symptom onset to medical care varies widely, ranging from 1.7 h to 21 h, but most patients are usually present within 6 h (3). Our results showed that only 11.5% of PNIA were diagnosed within 6 h of the onset of clinical symptoms. PAIS diagnosed after 24 h of symptom onset were further analyzed and the results suggested nearly half of them (46.67%) presented to the hospital with symptoms that had persisted for more than 24 h. This indicated that among these cases of delayed diagnosis of PAIS, over 50% occurred during the “post-hospital” phase. This underscored that, in order to improve time to diagnosis and intervention, educating and training healthcare setting was just as important as educating and training layperson.

Sudden onset of focal neurologic deficit is the clinical feature of stroke (23). The clinical manifestations of AIS in children are related to age, involved arteries, etiology, environment and many other factors (3, 23–25). Seizures are the most common clinical symptom of NAIS (12). Our results also supported this conclusion and the vast majority presented focal seizures. Hemiplegia is rare in NAIS (26) and we found only 2 cases (1.92%) of NAIS presented significant reduction in limb movement. Unlike NAIS, PAIS had a diverse clinical presentation. Limb weakness, facial palsy, disorders of consciousness, and seizures were the main initial neurologic symptoms of PAIS. In particular, limb weakness was the most common neurologic symptom in PAIS across age groups. The incidence of seizures in PAIS was 14.42%, which was significantly lower than in NAIS and occurred mainly in the group of children under 3 years of age. As age increases, PAIS seem to exhibit more other neurologic symptoms, including headache neurologic-related symptoms, including headache, vomiting, aphasia or dysarthria, unsteady gait, blurred vision and ptosis. The results supported that age was an important factor in differences in the clinical presentations of PAIS. However, we also need to be aware of the impact of differences in children’s own expressive language abilities at different ages and the altered levels of consciousness due to the stroke itself.

A retrospective study of perinatal ischemic stroke conducted by Machado et al. showed that the stroke site in NAIS was mostly on the left side (27). Data from the Canadian Pediatric Ischemic Stroke Registry showed there was more left-sided only involvement than right-sided only in NAIS (47% vs. 32%), but in PAIS, they were similar (40% vs. 39%) (20). Our results also showed that left-sided infarctions were higher than right-sided in NAIS. However, it was not evident in PAIS. An international multicenter observational study showed that there was more anterior circulation AIS than posterior circulation AIS in both NAIS and PAIS, with anterior circulation AIS accounting for 70% (28). Our results also yielded consistent conclusions and the NAIS group had a higher rate of infarctions that solely involved the cortex than PAIS, while infarctions in PAIS more commonly affected the basal ganglia region. Although we observed an improvement in ASPECTS or DWI- ASPECTS scores with age, the values indicated no significant difference among different age stages.

Risk factors for stroke in children are more complex than in adults and include heart disease, extracranial or intracranial arterial disease, thrombotic disease, SCD, systemic etiologies, and so on (3, 6, 29). Adult stroke risk factors such as atherosclerosis, hypertension, hyperlipidemia, alcoholism and smoking are rare in children. The etiology of stroke in children varies by race, e.g., SCD, a hemoglobin disorder that predominantly affects US children and children of African descent, is one of the most common arteriopathies associated with stroke in children (23), while none of SCD was found in a risk factors analysis of PAIS in China conducted by Wang et al. (30). Xie et al.’s investigation also found no evidence of SCD among risk factors for children with stroke, and the main risk factors were minor head injury, followed by moyamoya disease, viral encephalitis (5). We also found that in the PAIS group minor head injury was the most common risk factor, followed by heart disease, cerebrovascular malformations (including moyamoya disease) and intracranial infection. Also, we found that minor head injury related PAIS mainly occurred in children under 3 years old and decreased with age and MRI was interrogated for dissection in patients with minor head injury, but no evidence was found. Dowling et al. reported that nearly one-third of children with AIS had Cardiac disorders (31). However, Xie et al. reported that the incidence of heart disease in PAIS was only 5.0% (5). Our results showed that 13.46% of PAIS had heart disease, between the two reported incidence rates. Multiple factors interact to increase the risk of developing AIS. A retrospective surveyed by Lee et al. (32) found that 22% of PAIS had more than one risk factor. Our results were 10.58% of PAIS had at least 2 risk factors. In addition, our results showed that 21.15% of PAIS risk factors were undefined, which was very close to the result of 21.7% reported by Xie et al. (5). Compared to PAIS, the risk factors for NAIS are significantly different, which are often associated with fetal distress or hypoxia (27). Our results supported this view, suggesting that perinatal hypoxia or asphyxia was the most common possible risk factor for NAIS.

We retrospectively reviewed population characteristics, clinical manifestations, neuroimaging findings and risk factors analysis in childhood AIS. The limitation of this retrospective study is the incompleteness of some data due to the long-time breadth of the survey and the retrospective design, especially in the analysis of risk factors, which makes it difficult to be precise. Such as, almost all children with AIS do not have information on genetic testing, and we are unable to assess the incidence of genetically related etiologies such as adenosine deaminase 2 deficiency in childhood AIS. In addition, due to the improved performance and development of MRI equipment itself, the diagnosis of some risk factors such as cerebrovascular abnormalities may be underestimated in early patients. Our study also found that some patients had no obvious clinical manifestations, suggesting that the actual incidence of AIS in children may be underestimated. Given the retrospective nature of our study, certain limitations have to be considered including changes in neuroimaging modality and quality impacting diagnostic accuracy. Future prospective studies are needed to explore our hypothesis generating findings including the incidence rates and etiologies.

## Conclusion

AIS in children is male-predominant, and there is significant diagnostic delay in both NAIS and PAIS. NAIS and PAIS differ in clinical and imaging manifestations, and risk factors. Seizures were the most common initial neurologic symptom. Limb weakness was the most common initial neurological symptom in PAIS. Perinatal hypoxia or asphyxia was the most common possible risk factor in NAIS, and minor head injury was the most common risk factor in PAIS. Notably, we also observed that the actual prevalence of AIS in children, and the diagnosis of certain risk factors, may be underestimated. Education and training will be required both in the layperson as well as healthcare setting to improve time to diagnosis and intervention and ultimately outcomes for childhood AIS survivors. Given the limitations of retrospective studies, future prospective studies are required to confirm the hypothesis generated from this and other retrospective studies.

## Data Availability

The original contributions presented in the study are included in the article/supplementary material, further inquiries can be directed to the corresponding authors.
